# Trace Elements in Post-Mortem Tissues: A Review of Current Evidence and Forensic Challenges

**DOI:** 10.3390/toxics13090743

**Published:** 2025-08-31

**Authors:** Claudia Trignano, Angela Sabalic, Andrea Pisano, Davide Tutedde, Pablo Hernández-Camarero, Raffaele La Russa, Macarena Perán, Roberto Madeddu

**Affiliations:** 1 Department of Biomedical Science, University of Sassari, 07100 Sassari, Italy; ctrignano@uniss.it (C.T.); pisanoandrea87@gmail.com (A.P.); d.tutedde@phd.uniss.it (D.T.); rmadeddu@uniss.it (R.M.); 2 International Society for Research on Cadmium and Trace Element Toxicity (ISRCT), 07100 Sassari, Italy; 3 Division of Thoracic Surgery, European Institute of Oncology (IEO), 20141 Milan, Italy; 4 Department of Health Sciences, University of Jaén, Campus de las Lagunillas, E-23071 Jaén, Spain; phernand@ujaen.es (P.H.-C.); mperan@ujaen.es (M.P.); 5 Biopathology and Regenerative Medicine Institute (IBIMER), Centre for Biomedical Research, University of Granada, E-18016 Granada, Spain; 6 Department of Clinical Medicine, Public Health, Life Sciences, Environmental Sciences, University of L’Aquila, 67100 L’Aquila, Italy; raffaele.larussa@univaq.it; 7 Excellence Research Unit “Modeling Nature” (MNat), University of Granada, E-18016 Granada, Spain; 8 National Institute of Biostructures and Biosystems (INBB), 00136 Rome, Italy

**Keywords:** trace elements, heavy metals, autopsy, postmortem analysis, forensic toxicology, ICP-IMS, postmortem samples, forensic investigation

## Abstract

Background: Trace elements and heavy metals can provide valuable forensic information for individual identification, lifestyle reconstruction, and association with the scene or time of death and may also assist in linking objects to criminal activities. However, the lack of standardized guidelines and post-mortem reference values represents a significant limitation in forensic investigations. Methods: This review was conducted in accordance with the PRISMA statement. We performed a comprehensive literature study over the last ten years focusing on the analysis of trace elements and heavy metals in post-mortem tissues. Results: The search results from the databases yielded 247 records. The screening, according to PRISMA criteria, allowed us to select and include 19 articles. The results showed the need for standardized guidelines and reference values. Although post-mortem trace element analysis shows high potential for forensic applications, substantial methodological heterogeneity persists. Some studies have proposed preliminary reference values for cadmium (Cd) in kidneys and mercury (Hg) in hair but validated post-mortem reference ranges remain largely unavailable. Conclusions: The current literature demonstrates the forensic potential of trace element and heavy metals analysis including Cd, Hg, lead (Pb), Manganese (Mn), Aluminum (Al), Copper (Cu), Zinc (Zn), Iron (Fe), Thallium (Tl), Polonium (^210^Po) but also underlines the urgent need for standardized protocols and validated post-mortem reference values to improve interpretability and reliability in forensic contexts.

## 1. Introduction

Essential trace elements, which account for approximately 1% of human body mass, play a fundamental role in sustaining physiological functions; among them are Zinc (Zn), Copper (Cu), Iron (Fe), Cobalt (Co), Chromium (Cr), Manganese (Mn), and Selenium (Se) [1]. They act as structural components or cofactors for numerous enzymes, contributing to immune system function, gene expression, redox balance, cellular respiration, and the prevention of oxidative stress [2,3,4]. Although these elements are essential for human health, their imbalance may promote the onset of various types of cancer, neurodegenerative diseases, and other chronic conditions. Conversely, elements such as Lead (Pb), Cadmium (Cd), and Mercury (Hg) have no known biological role in human cells and are considered toxic even at low blood concentrations—specifically, levels exceeding 10 μg/dL for Pb and Hg and 5 μg/L for Cd [5,6].

In forensic science, the knowledge and application of trace element and heavy metal analysis can provide valuable information for linking individuals, objects, and locations, thereby assisting experts in victim characterization such as lifestyle reconstruction, association with the scene of death, estimation of the post-mortem interval or investigation of potential toxicological causes of death [7,8,9,10].

Moreover, these analyses can support the identification of criminally relevant exposures, as well as intentional poisoning or chronic environmental contamination [9]. Despite the recognized value of these elements analysis in forensic and toxicological investigations, the interpretation of these findings often remains challenging due to the lack of standardized guidelines and reference values. This gap complicates the differentiation between physiological background levels and pathological or toxicologically relevant concentrations, increasing the risk of misinterpretation in forensic contexts. Additionally, post-mortem redistribution processes, matrix selection (e.g., blood, liver, kidney, bone), and analytical variability further contribute to the complexity of interpreting post-mortem trace element and heavy metals concentrations [9,11].

To date, the understanding of post-mortem metal degradation within tissues remains limited, underscoring the urgent need for further studies aimed at establishing reliable reference ranges applicable in forensic practice.

In this review, we provide an updated overview of the current state of knowledge about the analysis of trace elements and heavy metals in postmortem tissues, regardless of the cause of death. Through this preliminary work, we aim to outline a research pathway that, with the support of future studies, may contribute to the definition of post-mortem reference concentrations applicable in forensic practice.

## 2. Methods

This review was conducted in accordance with the PRISMA statement [12]. A comprehensive literature search was performed in PubMed and Scopus. The last search was performed in May 2025. Only original full-text research articles published in peer-reviewed journals, in English, and within the last ten years were considered eligible.

The following search terms were used in various combinations: heavy metals AND autopsy, heavy metals AND postmortem, heavy metals AND autopsy AND postmortem, trace elements AND autopsy, trace elements AND postmortem, trace elements AND postmortem, trace element AND autopsy AND postmortem.

All studies evaluating the concentrations of heavy metals and trace elements in postmortem samples derived from autopsies were considered for inclusion. Specifically, we included original research articles and case series that reported quantitative data. We excluded individual case reports, conference abstracts, reviews, and studies not related to forensic analysis of human post-mortem samples.

**Figure 1:**
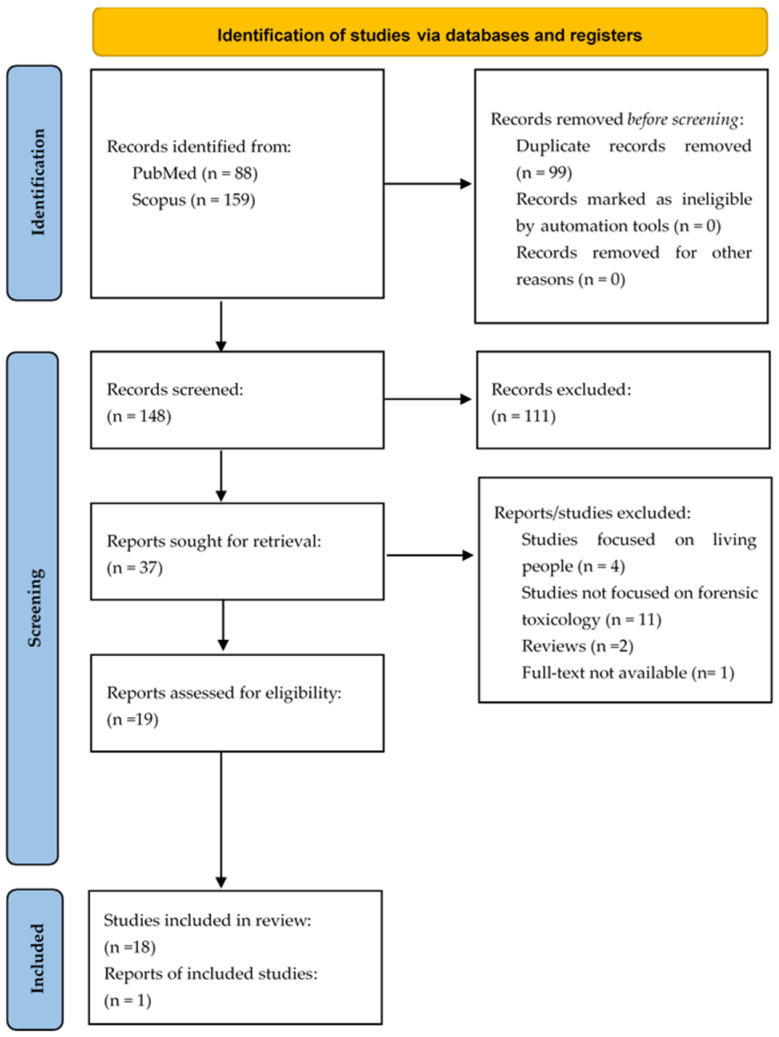
PRISMA flow diagram.

To ensure a PRISMA-guided selection process was applied, beginning with the removal of duplicates. Two authors then conducted screening in three consecutive phases: an initial review of titles, a second review of abstracts, and finally, a full-text assessment for eligibility. Each of these phases was performed independently by two authors. Moreover, article selection was managed using Microsoft Excel, which was used to track changes and compile the final database of included studies. Any discrepancies found at each stage were resolved through discussion and consensus. The study selection process is illustrated in the PRISMA flow diagram (Figure 1).

## 3. Results

The search results from the databases yielded 247 records; titles and abstracts of 148 total records were accessed in the screening phase, from which only 36 articles were selected for eligibility criteria. Finally, only nineteen studies (Table 1) were considered eligible for inclusion for full text-level review. The results were presented by answering the PEO (Population, Exposure, Outcome) Questions.

Xu et al. (2015) [13] analyzed tissue samples from 163 deceased individuals following trauma, comparing 120 patients who died between the 5th and 15th day of hospitalization with 43 who died immediately after severe traumatic brain injury. The concentrations of Cu, Fe, Zn, and Se were analyzed using ICP-AES in the heart, liver, brain, and kidneys. The authors observed a significant reduction in the levels of all four elements in the patients who died during hospitalization, highlighting the key role of these elements in trauma-related deaths and suggesting that their depletion may increase the risk of mortality.

Ramos et al. (2016) [14] analyzed brain tissue samples from 42 individuals without neurological or psychiatric disorders to investigate the distribution of alkali metals across 14 different brain regions. Using FAES and ICP-MS, the authors measured concentrations of Na, K, Li, Rb, and Cs, and observed a heterogeneous distribution, particularly for K, Li, and Cs, with the highest levels generally found in the putamen and the lowest in the brainstem and cerebellum. Rb and Na were more evenly distributed. No sex-related differences were found, but higher levels of K, Rb, and Cs were detected in smokers. This study provides baseline data on alkali metal content in the healthy human brain and emphasizes the need to consider regional variability when investigating metal alterations in neurological diseases.

Lech and Sadlik et al. (2017) [15] determined Cd concentrations in various organs, such as the brain, stomach, small intestine, liver, kidneys, lungs, and heart collected from 150 individuals aged between 1 and 80 years. The highest Cd levels were found in the kidneys (up to 61.3 µg/g), followed by the liver and intestine, while the heart showed lower concentrations. The study provides updated reference values for Cd, useful in distinguishing physiological levels from those due to chronic or acute exposure. In 2019, the same author [16] optimized and validated TDA-AAS (Thermal Decomposition, Amalgamation, and Atomic Absorption Spectrometry) for Hg quantification in forensic analyses. They used certified reference materials (CRM) and 100 post-mortem tissues (including blood, urine, and hair). The method proved accurate, precise, and reliable, outperforming traditional methods in terms of speed, simplicity, and elimination of sample preparation, making it suitable and recommended for forensic toxicological investigations of mercury.

Danuta and colleagues (2018) [17] assessed Cr concentrations in a population of 21 females and 39 males from Poland who died of causes unrelated to known environmental or occupational Cr exposure. The analysis was performed using Electrothermal Atomic Absorption Spectrometry on blood samples and other autopsy specimens, such as brain, stomach, liver, kidneys, lungs, and heart. The lungs and liver showed higher Cr concentrations. Statistically significant differences were also observed by sex (higher in males) and with increasing age. The study provides reference interval data for non-exposed populations.

Grochowski and colleagues (2019) [18] evaluated the post-mortem levels of Al and Si in 31 individuals with alcohol use disorder (AUD) and 32 control subjects. The analysis was performed in 10 brain regions and the liver using ICP-OES. Results showed a significant increase in Al in the AUD brain, particularly in regions associated with cognitive functions such as the frontal thalamus (FTH), inferior longitudinal fasciculus (ILF), and frontal insula (INS), while Al was undetectable in controls. Si was also significantly higher in the AUD brain vs. controls (*p* < 0.001), but with limited regional differences. No difference in Si was found in the liver. The authors suggest the potential neurotoxic effects of Al and its implication in neurodegenerative processes.

Ogunfowokan and colleagues (2019) [19] measured Cd, Pb, Mn, Cu, and Zn in urine from 35 patients and in 18 cadaver tissues. AAS revealed higher concentrations of Cu in the urine samples for both male and female patients than those of Cd, Pb, Mn and Zn, and also higher than the recommended standard level in human urine, especially older males. Post-mortem analysis showed Mn was highest across all organs, while Pb had the lowest concentrations. Findings confirmed the association between heavy metal accumulation and observed health complications in patients.

Di Candia and colleagues (2020) [20] reported a rare case of acute thallium (Tl) poisoning involving eight members of the same family in Italy, three of whom died. The intoxication was caused by voluntary adulteration of an herbal infusion with thallium sulfate by a family member. Post-mortem examinations were performed on the deceased (father, mother, and daughter), and biological samples were analyzed using ICP-MS. Elevated thallium concentrations were detected in blood, urine, gastric content, and hair. Hair segmental analysis confirmed repeated exposure over two months. Toxicological and forensic findings, combined with police investigations, led to the identification and confession of the perpetrator.

García, F. et al. (2020) [21] assessed the levels of 11 toxics and trace elements (As, Be, Cd, Cr, Hg, Mn, Ni, Pb, Sn, Tl, V) in kidney, liver, brain, bone, and lung tissues from 20 individuals who had lived for at least 10 years near the Constantí hazardous waste incinerator, without occupational exposure. Concentrations were measured by ICP-MS. Cadmium and lead accumulated predominantly in kidney and bone, respectively, while manganese levels were relatively high in kidney and liver. Arsenic, beryllium, thallium, and vanadium (V) were below detection limits in all samples; mercury was rarely detected. Over the past 20 years, significant decreases in Cd, Hg, and Pb levels were observed, likely due to environmental regulations (e.g., leaded gasoline ban), whereas chromium increased in kidney and bone. The authors demonstrate that no inorganic element bioaccumulation could be attributed to incinerator emissions.

Gunawardena et al., 2020 [22] have investigated the bioaccumulation of six elements comprising both trace elements (Cr, Zn, Se) and (Cd, As, Pb). The cohort included 13 urban and 18 rural cases. Significantly higher levels of Cd, Zn, and Se were found in urban residents. Zn and Se levels negatively correlated with age. As reported by the authors, these findings suggest rural populations may be more vulnerable to chronic kidney disease of uncertain etiology (CKDu) due to lower protective element levels. The same authors in 2021 analyzed kidney concentrations of Cd using ICP-MS in a population of 92 individuals from an urban area in Sri Lanka, excluding all deaths due to occupational exposure, poisoning, or chronic kidney disease. The mean Cd concentration for the entire population was 4.38 μg g^−1^, well below the estimated toxic threshold value (50 μg g^−1^). No statistically significant differences were observed by age or sex. This study excludes a potential environmental Cd exposure risk for the Sri Lankan population [23].

Fleischer et al. (2021) [24] focused on the systemic accumulation of metals in periprosthetic tissues of individuals with hip or knee implants. Using a semi-automated system with robotic preparation and ICP-MS analysis, high levels of Cr, Co, Ni, Ti, and Al were detected. The methodology was shown to be precise, reproducible, and efficient, providing a reliable tool for identifying metallosis or prosthetic malfunction in forensic settings.

Baj et al. (2022) [25] compared liver concentrations of 11 trace elements in 39 individuals with AUD and 45 controls, using ICP-OES on post-mortem samples. AUD cases showed significantly lower levels of Co, Cu, Mg, and Mn, with only Fe elevated. The study highlights disrupted hepatic trace element homeostasis in chronic alcohol consumers, with Fe possibly affecting inter-element dynamics. In the same periods they analyzed 51 elements in meningeal samples of 20 trauma victims and 20 suicide using tandem ICP-OES. The authors find significant differences concerning mainly metals from the lanthanide family (Ln), macroelements (Na, K, Ca, Mg), a few micronutrients (Co), and toxic Cd. The performed evaluation of the element distribution in the human meninges sheds new light on the inorganic element metabolism in the central nervous system, although we do not yet fully understand the role of the human meninges [26].

Sahar Y. Issa et al. (2022) [27] analyzed 400 autopsy cases with causes of death unrelated to heavy metal poisoning and 400 blood and urine samples from living individuals, with the aim of comparing levels of eight metals (Pb, Cd, Cu, Zn, Fe, Se, Mn, and Cr) in various biological samples to enable proper interpretation of forensic toxicological results. Metal levels tended to be higher in cardiac blood compared to femoral blood, and post-mortem samples often showed higher levels than those from living individuals, especially for toxic elements.

Villa dos Santos and colleagues (2022) [28] analyzed lung tissues from 20 São Paulo residents to assess metals and Polonium-210 (^210^Po) due to urban air pollution. Neutron activation analysis identified associations between ^210^Po and vehicular emissions. Regression showed a significant link between ^210^Po and traffic-related metal exposure. The study supports using lung tissue metal content as a marker of long-term urban pollution exposure.

Baj et al. (2023) [30] analyzed postmortem brain and liver samples from 39 individuals with atherosclerosis (ATH), steatosis, both, or no pathology. Using ICP-MS, they analyzed 51 elements. The results obtained allow us to conclude that the hepatic steatosis group suffers from a deficiency of important trace elements, such as Cu, Zn, and Mo, whereas the group with atherosclerosis is characterised by elevated levels of Cd in the liver tissue. In addition, an accumulation of Cd, Pb, Ti, and Sr in the brain tissue was observed in the atherosclerosis group. In addition, the disruption of trace elements homeostasis in the brain of a single case with bipolar disorder, and a case with hip replacement was observed. The authors confirm the involvement of chemical elements in the pathogenesis of selected metabolic diseases.

Ćirović et al. (2024) [29] analyzed postmortem left ventricular myocardium samples from 19 individuals with secondary cardiomyopathy (CMP) and 33 controls. Using ICP-MS, they found significantly higher levels of lead, nickel, manganese, and copper in CMP subjects, while zinc was significantly lower. No differences were observed for As, Cd, Hg, Fe and Mg. Negative correlations between Zn and several cardiotoxic metals suggest a role of trace metal imbalance in CMP pathogenesis.

Erkman and colleagues (2025) [31] examined postmortem blood from 70 suicide cases and 38 controls. ICP-MS showed higher levels of Cu, Zn, Mo, Co, and As, in suicides. Urban residence correlated with higher Cd and Hg. Gender differences emerged, with women showing higher Cu and Co. The study proposes metal screening as a useful tool in suicide risk assessment.

## 4. Discussion

This review aims to understand the state of the art regarding the use of trace elements and heavy metals in forensic practice to lay the groundwork for defining post-mortem reference concentrations applicable in this field. Although essential and toxic elements provide valuable forensic information, there is still a significant gap in distinguishing physiological from pathological levels in post-mortem tissues.

The included studies primarily examined post-mortem tissues obtained during autopsies, including individuals with alcohol use disorder (AUD) [18,25], suicide victims [29,31], trauma victims [13,30], individuals with secondary cardiomyopathy [30], and those exposed to environmental pollutants [21,22,23,28]. Additionally, some studies focused on forensic cases of acute poisoning [20] or patients with prosthetic implants [24]. Several works included control groups of healthy individuals or individuals without known neurological, psychiatric, or toxicological conditions [14,15,17,27]. It should be noted that some comparative studies included samples from living individuals, used to establish baseline values or to highlight post-mortem changes in metal concentrations [19,27]. The most interesting information found in the analyzed manuscripts concerns the following metals, Cd, Pb, Mn, Al, Cu, Zn, and Fe, and other metals like Na, K, Li, Rb, and Cs, Tl, ^210^Po.

### 4.1. Cadmium

Cd stands out as one of the most studied elements, with particular attention to its toxicity and accumulation. Lech, T. et al. [15] as well as García, F. et al. [21] reported the highest Cd concentrations in kidney and urine samples, compared to other organs such as the intestine, liver, lungs, and heart. Conversely, Ogunfowokan and colleagues observed renal Cd concentrations lower than those of Mn [19]. Gunawardena (2021) [23], comparing rural and urban populations in Sri Lanka, found levels well below the toxic threshold (50 μg g^−1^), thus excluding a significant risk of environmental exposure in that specific area [24]. Baj et al. (2022) [26] observed altered Cd/Zn, Cd/Cu, and Cd/Se ratios, suggesting an alteration of metal homeostasis and potential neurotoxic interactions [25]. These findings support the use of cadmium as an indicator of chronic exposure and potential metabolic dysfunction but highlight the need to distinguish between toxic and non-toxic accumulation across different tissues.

### 4.2. Lead

Pb is another widely studied metal, known for its neurotoxicity. Garcia et al. [21] highlighted that Pb predominantly accumulates in bones and reported a significant decrease in Pb levels over the last 20 years, likely due to environmental regulations such as the ban on leaded gasoline. In contrast, Ćirović et al. [29] observed significantly higher Pb levels in the left ventricular myocardium of individuals with secondary cardiomyopathy, suggesting a role for lead in the pathogenesis of this condition. Issa et al. [27] compared lead levels in post-mortem samples and living individuals, finding that post-mortem samples often showed higher levels of toxic elements like Pb. Overall, lead remains a reliable marker of chronic environmental exposure, with potential implications in the pathogenesis of cardiovascular and neurological disorders. 

### 4.3. Manganese

Mn has shown variable distribution and roles depending on tissue type and clinical condition. Garcia et al. (2020) [21] reported relatively high Mn levels in the kidneys and liver compared to brain, bone, and lung. Ćirović et al. (2020) [29] found significantly higher Mn levels in the myocardium of subjects with cardiomyopathy. Baj et al. (2022) [25,26] reported significantly lower Mn levels in the liver of individuals with AUD, suggesting a dysregulation of hepatic trace element homeostasis. Ogunfowokan and colleagues [19] found that post-mortem Mn levels were the highest among all post- mortem organs examined, emphasizing the influence of lifestyle and environment on metal accumulation. Finally, Baj et al. [30] observed increased hepatic accumulation of Mn in trauma victims, providing baseline data for post-mortem metallomics. The variability in manganese levels emphasizes the need for context-based interpretation, considering both the tissue examined and the clinical background.

### 4.4. Aluminum

Al has been specifically associated with neurological disorders. Grochowski et al. [18] found significantly elevated Al levels in the brains of individuals with AUD, particularly in cognitive regions like the frontal thalamus, inferior longitudinal fasciculus, and frontal insula, while Al was undetectable in controls. The authors suggested potential neurotoxic effects of Al and its implication in neurodegenerative processes. Fleischer et al. [24] detected high Al levels in periprosthetic tissues of individuals with hip or knee implants, indicating systemic accumulation in metallosis contexts. Baj et al. [30] also noted the accumulation of Al in the thalamus of trauma victims. The accumulation of aluminum in critical brain areas and in prosthesis-associated tissues suggests a potential neurotoxic and neurodegenerative role relevant to forensic investigations.

### 4.5. Copper, Zinc, Iron

Cu, Zn, and Fe are essential trace elements for numerous biological processes; however, even slight fluctuations in their concentrations can induce metabolic and immune dysfunctions. According to Baj, J. et al., positive correlations among metal concentrations were predominant in AUD livers compared to controls. Specifically, Mg showed strong positive correlations with Ca, Mn, and Fe; K was positively correlated with Mn and Zn, while Cu showed positive correlations with both K and Zn. Ćirović et al. [29] found significantly higher Cu levels and lower Zn levels in the myocardium of subjects with cardiomyopathy, with negative correlations between Zn and cardiotoxic metals. Xu et al. [13] observed significantly reduced levels of Cu, Fe, and Zn in the heart, liver, brain, and kidneys of patients who died during trauma hospitalization, suggesting a key role of these trace elements in trauma-related deaths. Erkman et al. [31] found higher levels of Cu and Zn in post-mortem blood samples from suicide cases, with gender differences. The complex interactions among Cu, Zn, and Fe make it difficult to define post-mortem reference ranges, but their analysis can reveal systemic imbalances relevant in chronic diseases, trauma, or suicide cases.

### 4.6. Other Metals

Other metals, such as the alkali metals Na, K, Li, Rb, and Cs, have been studied by Ramos et al. (2016) [14], who observed a heterogeneous distribution in the brain, and by Baj et al. [26], who found lower alkali levels in the meninges of suicide victims. In particular, metals like Tl were reported by Candia and colleagues in a case of acute mass poisoning, while ^210^Po was linked to urban air pollution by Villa dos Santos et al. [28]. These lesser-studied metals may serve as important markers in specific cases of acute or environmental exposure, reinforcing the need to include uncommon elements in forensic profiles. A limited number of the included studies attempted to define preliminary reference values, for example, for Cd in the kidney [15,23], Hg in hair [16], and Cr in the stomach and lungs [17]. In other cases, autopsy analysis proved more useful for correlating metal concentrations with the onset of specific pathologies [30] or for identifying them as markers of chronic environmental exposure [27].

Although these findings provide valuable insights into the accumulation and distribution of metals and trace elements in human tissues, the high heterogeneity and variability in experimental design and analytical approaches—such as ICP-MS, ICP-OES, FAES, AAS, and TDA-AAS—complicate cross-study comparisons and highlight the need for harmonized procedures and validated reference ranges. Supporting this, some studies have also addressed the validation and forensic applicability of the analytical techniques themselves [16,20,24,27].

Another crucial aspect of forensic analysis is the phenomenon of post-mortem redistribution [31]. While PMR has been extensively studied in relation to drugs and substances of abuse, scientific evidence suggests that it is equally relevant for heavy metals and trace elements [32,33]. The susceptibility of a substance to post-mortem redistribution primarily depends on its physicochemical properties but can also be influenced by factors such as temperature, cadaver storage conditions, post-mortem interval (PMI), and sampling site.

Among the analyzed studies, that of Issa et al. [27] provides particularly relevant results: the authors observed significant differences in the concentrations of several elements between deceased individuals and living subjects. Cadmium (Cd) and zinc (Zn) showed higher blood concentrations compared to other organs, with cadmium displaying a high central-to-peripheral blood ratio. Additionally, a study conducted on blood samples taken from various anatomical sites (heart ventricle, veins, and arteries) in cases of suspected intoxication showed that in deaths with a short PMI, no significant differences were observed between central and peripheral blood. In contrast, in cases with a medium-to-long PMI, concentration ratios were significantly increased [34]. Detected concentrations can also be influenced by intrinsic factors such as genetic predisposition, tolerance to the substance, comorbidities, lifestyle, and environmental exposure. For example, Cd blood levels are reported to range between 0.4–1 µg/L in non-smokers and 1.4–4 µg/L in smokers globally [35].

Occupational or environmental exposure can result in much higher levels: in Pakistan, a Cd concentration of 373 µg/L was recorded in exposed individuals compared to 22 µg/L in non-exposed subjects [36]. In contrast, Gunawardena [23], comparing rural and urban populations in Sri Lanka, found values well below the toxic threshold (50 µg/g), excluding a significant environmental risk in that area. Conversely, Villa dos Santos et al. [28] detected elevated levels of 210^Po in the lung tissue of individuals residing in São Paulo, suggesting a possible correlation with local air pollution.

These findings highlight the complexity of post-mortem toxicological interpretation, particularly concerning heavy metals and trace elements. Their distribution in tissues is influenced by multiple environmental, biological, and post-mortem redistribution factors, requiring an integrated and multidisciplinary approach to ensure diagnostic accuracy and forensic value. The establishment of reliable reference values and the harmonization of analytical protocols are essential steps to improve comparability between studies and the reliability of toxicological results obtained from autopsy samples.

Although the search strategy was designed to be as comprehensive as possible and the results obtained are promising, this review presents some important limitations. Primarily, there was significant heterogeneity among the included studies, both in terms of experimental design and analytical approach. Variability in the techniques used (e.g., ICP-MS, AAS, FAES) and the lack of standardized protocols for sampling and analysis make direct comparison of results challenging. These factors necessitated a narrative approach. While this approach is useful for integrating and contextualizing heterogeneous information, it may be affected by the availability and quality of data reported in the analyzed studies.

Nevertheless, despite these limitations, we believe that this review offers a useful overview of the current state of knowledge and may provide new insights for future research in the field of forensic toxicology of metals and trace elements. To provide an interpretive aid for the criteria discussed, a detailed flowchart has been included in the Supplementary Materials (Figure S1).

## 5. Conclusions

Post-mortem trace element and metal analysis represents a valuable but underutilized tool in forensic investigations. It has the potential to contribute to exposure assessment, cause-of-death determination, and toxicological evaluation, particularly in cases involving suspected environmental or occupational exposures, poisoning, or unclear clinical histories. However, current methodological inconsistencies and interpretative challenges related to variables such as the post-mortem interval, preservation conditions, and sampling protocols limit its standardized forensic applicability. For this reason, future research should focus on addressing these critical issues in order to integrate this approach into routine forensic practice.

The evidence gathered in this review lays the groundwork for future quantitative and meta-analytic studies that, by integrating existing data, could contribute to the development of interpretative thresholds for specific trace elements—ultimately improving the reliability and forensic utility of these markers.

## Figures and Tables

**Table 1:** Summary Table of selected articles—The table summarizes the key elements from the analyzed articles [13,14,15,16,17,18,19,20,21,22,23,24,25,26,27,28,29,30,31]. Authors Baj et al., 2022 [26], and Baj et al., 2023 [30], analyzed 51 elements; in the table, we report only the significant ones.

Year	First Author	Title	Population	Samples	Metals	Analysis Metod	Ref.
2015	Xu, G. et al.	Trace Element Concentrations in Human Tissues of Death Cases Associated With Secondary Infection and MOF After Severe Trauma	Patients due to hospitalization (n = 120) Deaths immediately (n = 43)	Brain, Liver, Heart, Kidney	Cu, Fe, Zn, Se	ICP-AES	[13]
2016	Ramos, P. et al.	Alkali metals levels in the human brain tissue: Anatomical region differences and age-related changes	Subjects with no known neurological and psychiatric history disorder (n = 42)	Brain	Na, K, Li, Rb, Cs	FAES, ICP-MS	[14]
2017	Lech, T. et al.	Cadmium Concentration in Human Autopsy Tissues	Subjects aged between 1 and 80 years (n = 150)	Brain, Liver, Kidney, Stomach, Intestine, Heart, Lung	Cd	TDA-AAS	[15]
2018	Dudek-Adamska, D. et al.	Chromium in Postmortem Material	No worked exposed subjects (n = 60)	Blood, Brain, Stomach, Liver, Kidney,	Cr	EAAS	[17]
2019	Lech, T. et al.	Application of TDA AAS to Direct Mercury Determination in Postmortem Material in Forensic Toxicology Examinations	Subjects never poisoned and exposed (n = 75)	Blood, Urine, Hair, Bile, Vitreous humor	Hg	TDA AAS	[16]
2019	Grochowski, C. et al.	Increased Aluminum Content in Certain Brain Structures is Correlated with Higher Silicon Concentration in Alcoholic Use Disorder	Alcoholic Use Disorder (n = 31) Controls (n = 32)	Brain, Liver	Al, Si	ICP-MS	[18]
2019	Ogunfowokan, A.O. et al.	Determination of Heavy Metals in Urine of Patients and Tissue of Corpses by Atomic Absorption Spectroscopy	Patient (n = 35) Autopsy (n = 18)	Urine	Cd, Pb, Mn, Cu, Zn	AAS	[19]
2020	Di Candia, D. et al.	Thallium toxicity due to audultered infusion with thallium sulfate in eight members belonging to the same family nucleus: Autopsy findings and ICPMS analysis (inductively coupled plasma mass spectrometry) in a triple homicide	Family members poisoned (n = 8)	Cardiac blood, urine, Gastric Content, Hair	Tl	ICP-MS	[20]
2020	García, F. et al.	Biomonitoring of Trace Elements in Subjects Living Near a Hazardous Waste Incinerator: Concentrations in Autopsy Tissues	Subjects near the Waste Incinerator (n = 20)	Kidney, Liver, Brain, Bone, Lung	As, Be, Cd, Cr, Hg, Mn, Ni, Pb, Sn, Tl, V	ICP-MS	[21]
2020	Gunawardena, S.A. et al.	Renal bioaccumulation of trace elements in urban and rural Sri Lankan populations: A preliminary study based on post mortem tissue analysis	Subjects form urban district (n = 13) Subjects from rural district (n = 18)	Kidney	Cr, Zn, Se, Cd, As, Pb	ICP-MS	[22]
2021	Fleischer, H. et al.	Semi-Automated Determination of Heavy Metals in Autopsy Tissue Using Robot-Assisted Sample Preparation and ICP-MS	Subjects with hip or knee implants deceased (n= 5)	Brain, Heart, Lung, Kidney, Liver, Fatty, Bone	Cr, Co, Ni, Ti, Al	ICP-MS	[24]
2021	Gunawardena, S.A. et al.	Kidney Cadmium Concentrations in an Urban Sri Lankan Population: an Autopsy Study	No exposure subjects (n = 92)	Kidney	Cd	ICP-MS	[23]
2022	Baj, J. et al.	Chronic Alcohol Abuse Alters Hepatic Trace Element Concentrations-Metallomic Study of Hepatic Elemental Composition by Means of ICP-OES	Alcoholic Use Disorder (n = 39) Controls (n= 45)	Liver, Blood	Ca, Co, Cr, Cu, Fe, K, Mg, Mn, Na, Zn, Se	ICP-MS	[25]
2022	Baj, J. et al.	ICP-MS Multi-Elemental Analysis of the Human Meninges Collected from Sudden Death Victims in South-Eastern Poland	Suicide (n = 20) Subjects accident victims (n = 20)	Brain	Ln, Na, K, Ca, Mg, Co, Cd	ICP-OES	[26]
2022	Issa, S.Y. et al.	Estimation of blood and urine levels of eight metals and essential trace elements collected from living Subjects compared to urine, cardiac and femoral postmortem blood, and other postmortem samples: A forensic toxicology study	Deceased subjects (n = 400) Lived subjects (n = 400)	Blood, Urine, Spleen, Liver Kidney,	As, Se, Ag, Cd, Sb, Hg, Zn, Pb	ICP-MS	[27]
2022	Villa dos Santos, N. et al.	Accumulation of trace element content in the lungs of Sao Paulo city residents and its correlation to lifetime exposure to air pollution	Exposed subjects lifetime to air pollution (n = 20)	Lung	Br, Ca, Ce, Cl, Cr, Co, Cs, Fe, Hf, K, La, Mn, Na, Rb, Sb, Sc, Se. Th, Zn, ^210^Po	SEGe, DSA	[28]
2023	Baj, J. et al.	Linking Metallic Micronutrients and Toxic Xenobiotics to Atherosclerosis and Fatty Liver Disease—Postmortem ICP-MS Analysis of Selected Human Tissues	Subjects with various medical disorders (n = 39)	Liver, Brain	Ca, K, Na, Mg, Zn, Fe, Mo, Mn, Cu, Se, Cd, Hg, Bi, Al	ICP-MS	[30]
2024	Ćirović, A. et al.	Trace Element Concentrations in Autopsied Heart Tissues from Patients with Secondary Cardiomyopathy	Cardiomyopathy patients (n = 19) Controls (n = 33)	Heart	Fe, Zn, Cu, Mn, Ni, Mg, Cd, Pb, Hg, As	ICP-MS	[29]
2025	Erkman, F. et al.	Heavy metal levels in postmortem blood samples: Unraveling links to suicide and neurological impacts	Suicide victims (n = 70) Controls (n = 38)	Blood	Cr, Mn, Cd, Sb, Pb, Cu, Zn, Se, Mo, Co, As, Ni, Hg	ICP-MS	[31]

ICP-MS, inductively coupled plasma mass spectrometry; ICP-OES, inductively coupled plasma optical emission spectrometry; AAS, atomic absorption spectrometry (TDA = graphite furnace, EA = electrothermal); FAES, flame atomic emission spectrometry; SEGe/DSA, specific extraction/digestion and analysis.

## Supplementary Materials

The following supporting information can be downloaded at https://www.mdpi.com/article/10.3390/toxics13090743/s1, Figure S1: Framework for the Forensic Evaluation of Matrices.
