# Supplementary material for: Trace Elements in Post-Mortem Tissues: A Review of Current Evidence and Forensic Challenges
Source: Toxics. 2025 Aug 31;13(9):743. doi: 10.3390/toxics13090743 (PMC12473529; doi:10.3390/toxics13090743)

Figure S1. Framework for the Forensic Evaluation of Matrices. A flowchart summarizing the criteria for the selection of biological matrices based on the type of exposure (acute, chronic), ongoing pathology, or implanted prosthesis. This framework is intended as an interpretive aid and not a definitive decision-making tool, given the limited availability of studies and the partial nature of the reported clinical cases.

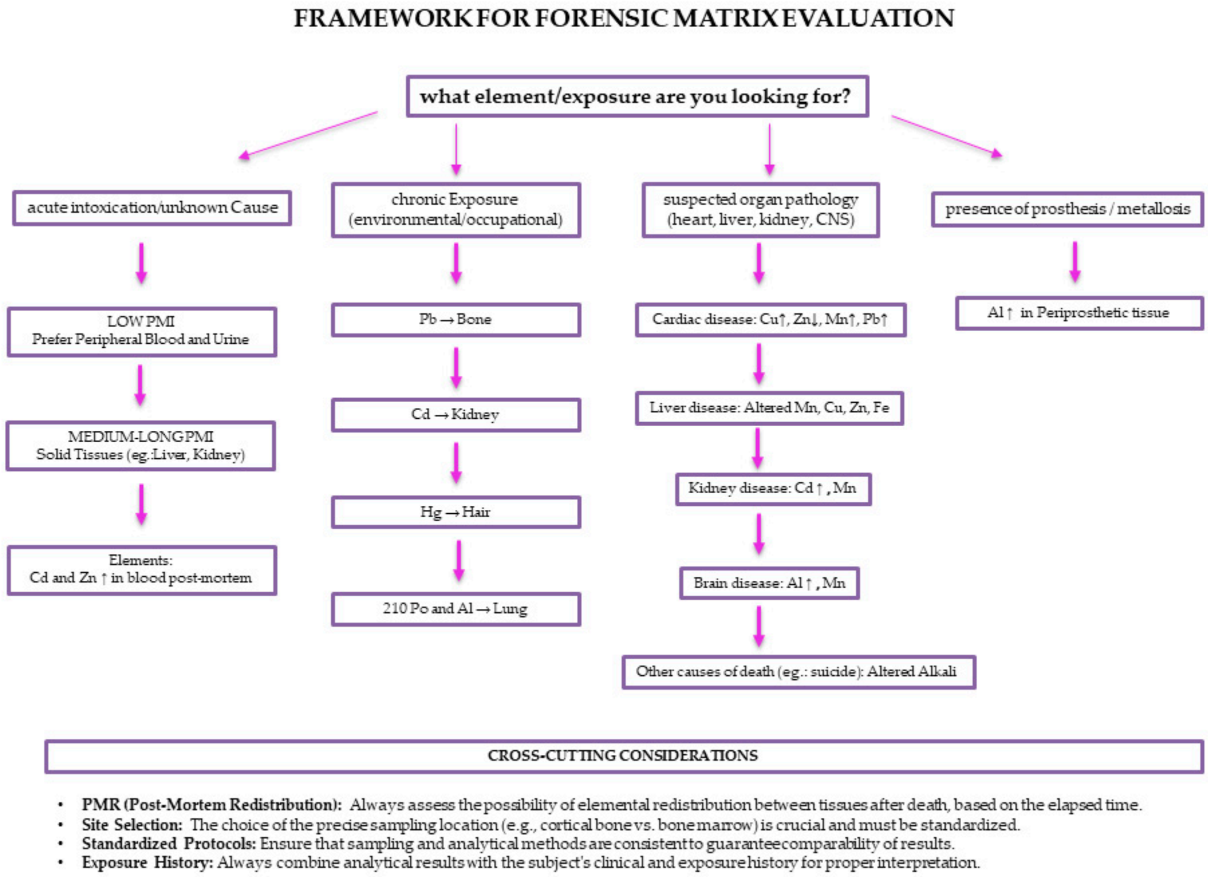

Supplement: Supplementary file 1 [file toxics-13-00743-s001.zip › toxics-3810033-supplementary.pdf]
